# Hematoscopy

**Published:** 1886-10

**Authors:** 


### Medical News.

HEMATOSCOPY.—Henrocque, of Paris, in a communication addressed to the French Academy of Sciences, gives the results obtained by his method of Hematoscopy. This is based upon the employment of the spectroscope for the analysis of blood, and comprises two series of examinations. First, the quantity of oxyhemoglobine is obtained by a spectroscopic examination of a few drops of blood drawn from the finger; second, the blood is examined by the spectroscope through the thumb nail; a ligature is applied and the time required for the reduction of the oxyhemoglobine is thus determined. Having obtained these two factors, viz.: the quantity of oxyhemoglobine, and the time required for its reduction in the tissues, he finds that the normal time required for the reduction of blood containing fourteen per cent. of oxyhemoglobine is seventy seconds. From these data the value of a unit of reduction is determined, which is fixed as the tissue activity required to reduce a solution of two per cent. of oxyhemoglobine in one second. Henrocque has made observations on 175 individuals, and finds that the activity of reduction varies in different constitutions and diseases independent of the quantity of oxyhemoglobine in the blood. Various medicines also retard or increase the activity of reduction. This method, which shows directly the rapidity of the consumption of oxygen in the tissues, opens up a new field for the study of nutrition, and particularly the changes going on between the tissues and the blood.—*Progres Med.*

---

Serebizki, in a paper recently read before the Russian Medical Congress, exhibited statistics showing that nearly one per cent. of all the men examined for the Russian armies are blind in one or both eyes. That is, of 1,388,761 men examined, 13,688 were blind. In addition to those found blind, 6,287 were affected with staphyloma, exophthalmos, symblepharon or leucoma; and 9,059 were rejected on account of asthenopia. In France, the statistics show that about one person in a thousand is blind. Blindness is therefore ten times more frequent in Russia than in France. Engineer Melville, who spent a winter in Siberia with the survivors of the "Jeannette," observed that there was scarcely a cabin in all Northern Siberia that did not contain one or more blind pensioners. He states that the majority of the inhabitants are syphilitic and that they infect their eyes by their peculiar method of ablution. Filling the mouth with water, they blow it upon their hands and then apply it to their faces. In this way Mellville explains the infection of the eyes, an explanation, however, that will scarcely satisfy medical men. The true cause of many of these cases of blindness is syphilitic iritis, a disease which seldom receives any treatment among the Russian peasantry. Cataract is also very frequent in the northern provinces, and is supposed to be due to snow blindness.

